# Author Correction: Uncovering mechanisms of global ocean change effects on the Dungeness crab (*Cancer magister*) through metabolomics analysis

**DOI:** 10.1038/s41598-023-30370-z

**Published:** 2023-04-05

**Authors:** Shelly A. Wanamaker, Paul McElhany, Michael Maher, Danielle Perez, D. Shallin Busch, Krista M. Nichols

**Affiliations:** 1 Division of Biological Sciences, Cell and Developmental Biology Section, University of California San Diego, La Jolla, CA USA; 2 Genomic Analysis Laboratory, The Salk Institute for Biological Studies, La Jolla, CA USA; 3 Conservation Biology Division, Northwest Fisheries Science Center, National Marine Fisheries Service, National Oceanic and Atmospheric Administration, Seattle, WA USA; 4 Ocean Acidification Program, National Oceanic and Atmospheric Administration, Seattle, WA USA; 5 Present Address: School of Aquatic and Fishery Sciences, University of Washington, Seattle, WA USA

Correction to: *Scientific Reports* 10.1038/s41598-019-46947-6, published online 24 July 2019

Since the publication of this work, Shelly A. Wanamaker has changed their name from Shelly A. Trigg.

The original HTML and PDF versions of this Article have been corrected.

